# Supplementary material for: Dispersion engineered metasurfaces for broadband, high-NA, high-efficiency, dual-polarization analog image processing
Source: Nat Commun. 2023 Nov 4;14:7078. doi: 10.1038/s41467-023-42921-z (PMC10625611; doi:10.1038/s41467-023-42921-z)
Supplement: Supplementary file 1 — Supplementary Information [file 41467_2023_42921_MOESM1_ESM.pdf]

Table of Contents

Section S1. Optical Characterization

Section S2. Additional numerical and experimental data

Section S.2.1 Transfer functions

Section S.2.2 Measured transmission amplitudes for $\phi = 30^o$

Section S.2.3 Edge detection with rectangular targets

Section S3. Image processing with metasurfaces – theoretical calculations

Section S4. Maximum achievable efficiency

Section S5. Origin of additional peaks in the output images

Section S6. Increasing the bandwidth

S1. Optical Characterization

The transmission amplitudes shown in Figs. 2(g-j) of the main text were performed with the custom-built setup shown in Fig. S1a. The sample was mounted on two different rotation stages, a motorized one (Thorlabs, HDR50) to control the polar angle θ, and a manual one to control the azimuthal angle ϕ. A broadband supercontinuum laser (NKT, SuperK) was filtered via a commercial narrowband filter (Photon, LLTF Contrast) and then injected into the setup via a fiber. The filtered laser has a linewidth of approximately 5 nm (see Fig. S1b). The laser was weakly focused on the metasurface via a lens (L1) with f = 20 cm focal length. The transmitted signal was collected and re-collimated on the other side of the sample by an identical lens (L2). Two identical germanium powermeters (Thorlabs, S122C), P1 and P2, were used to measure the transmission level through the metasurface. A beamsplitter (BS), placed before the excitation lens L1, was used to redirect approximately 50% of the laser power to the photodiode P2. A linear polarizer placed before the beamsplitter was used to polarize the incoming beam along either x or y, which correspond, respectively, to p- and s-polarization for any value of θ and ϕ. A second polarizer after lens L2 was used to select the polarization of the collected beam. The transmission amplitudes shown in Figs. 2(g-j) were then obtained by sweeping the angle θ and the input wavelength and recording the powers measured by P1 and P2. An additional calibration run was taken without the metasurface, to account for the exact splitting ratio of the BS and for discrepancies between the two powermeters.

The imaging experiments shown in Figs. 4 and 5 of the main paper were performed with the setup shown in Fig. 4a. The illumination was provided by the same supercontinuum source used in the setup in Fig. S1a. For the measurements in Fig 4(b-c), the broadband source was filtered by the

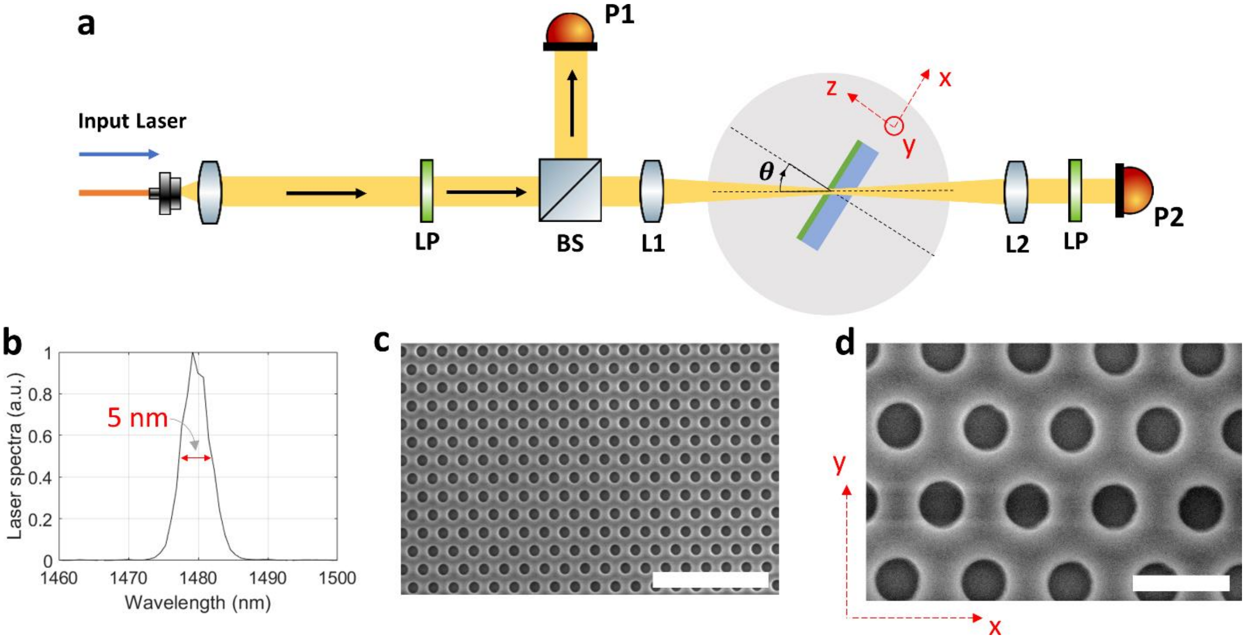

Figure S1. (a) Schematic of the setup used to perform the angle- and wavelength-dependent transmission measurements. LP = Linear polarizer, BS = beamsplitter, L1, L2 = lenses, P1, P2 = photodiodes. See text for additional details. (b) A representative spectrum of the laser used for the measurements shown in Figs. 2(g-j) and Fig. 4 of the main text, centered at 1480 nm. The laser has a linewidth of about 5 nm. (c-d) SEM pictures of the fabricated device, at different scales. Scale bars: 5 μm in panel c and 1 μm in panel d.

same narrow band filter used for the transmission measurements. For the measurements in Fig. 5, the output of the supercontinuum laser was filtered with a custom-built pulse shaper that allows to continuously tune the linewidth and central wavelength of the input spectrum.

S.2 Additional numerical and experimental data

S2.1 Transfer functions

In Fig. 3 of the main text we showed the absolute values of the co-polarized transfer functions, $|t_{ss}|$ and $|t_{pp}|$, for five different wavelengths. The full co- and cross-polarized complex transfer functions, for the same set of wavelengths, are shown in Figs. S2 and S3. As mentioned in the main text, the magnitude of the cross-polarized transfer functions $|t_{ps}|$ and $|t_{sp}|$ (Figs. S2c and S2d, respectively) are very small within the numerical aperture NA = 0.35 (dashed circles n in each plot). The phases of t_{pp} and t_{ss} (Figs. S3a and S3b, respectively) are quite uniform upon variation of the azimuthal angle ϕ, confirming the excellent isotropy of our devices. The phases are also fairly constant upon variations of the polar angle θ, except for jumps that occur when the corresponding amplitude becomes zero (compares Figs. S3(a-b) with Fig. S2(a-b)). The phases of the cross-polarized transfer functions (Figs. S3(c-d)) show a more convoluted behavior. However, due to the very low values of the corresponding magnitudes, they play a negligible role in the overall imaging.

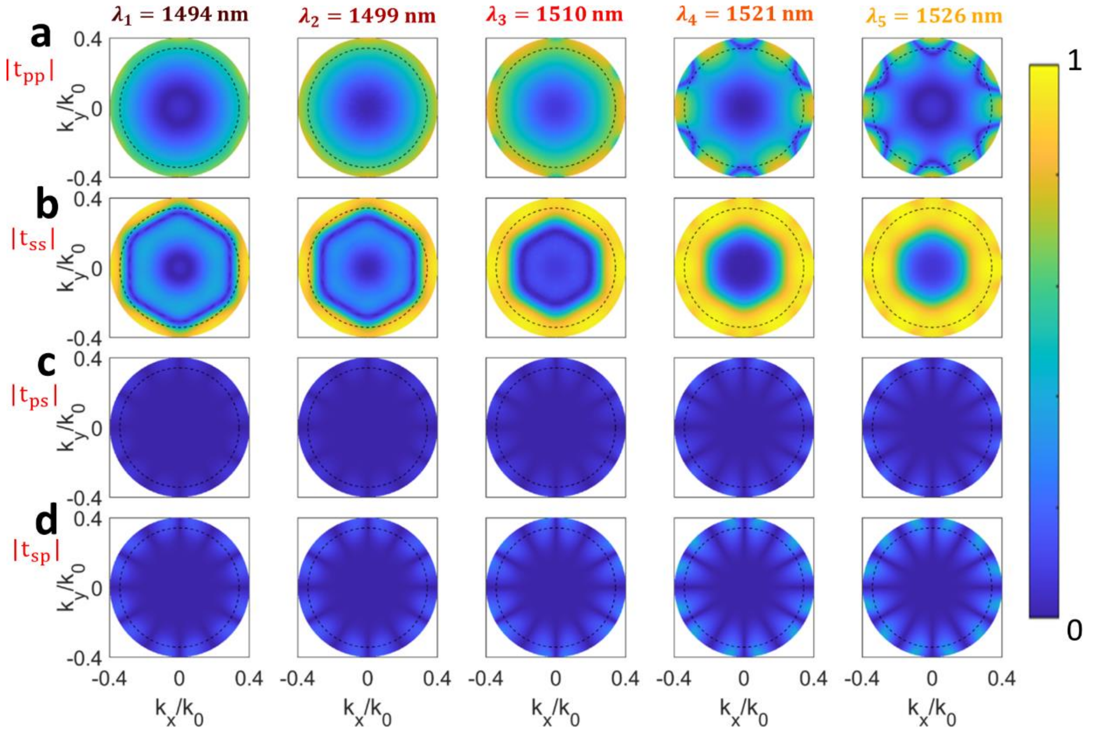

Figure S2. Calculated magnitude of the complex co- and cross-polarized transfer functions for the five wavelengths considered in Fig. 3 of the main text. Each column corresponds to a different wavelength, reported on top of the column. (a) Co-polarized transfer functions $|t_{pp}|$. (b) Co-polarized transfer functions $|t_{ss}|$. (c) Cross-polarized transfer functions $|t_{ps}|$. (d) Cross-polarized transfer functions $|t_{sp}|$.

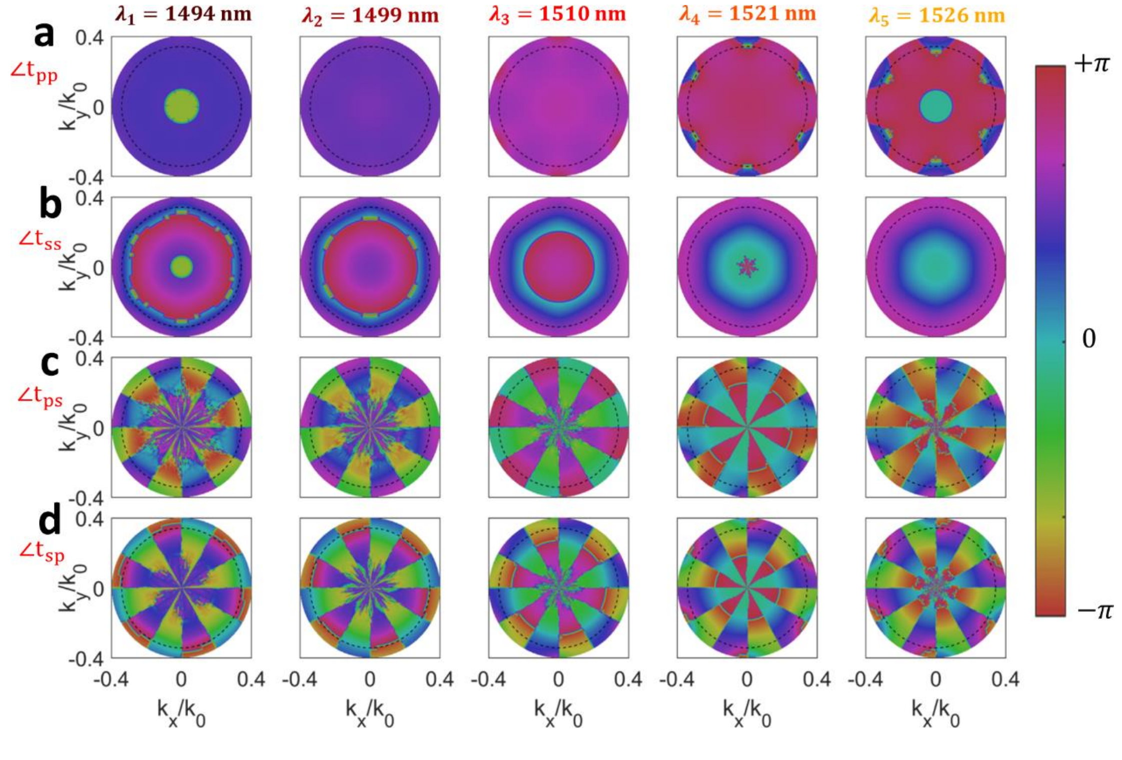

Figure S3. Calculated phases of the complex co- and cross-polarized transfer functions for the five wavelengths considered in Fig. 3 of the main text. Each column corresponds to a different wavelength, reported on top of the column. (a) Co-polarized transfer functions $\angle t_{pp}$. (b) Co-polarized transfer functions $\angle t_{ss}$. (c) Cross-polarized transfer functions $\angle t_{ps}$. (d) Cross-polarized transfer functions $\angle t_{sp}$.

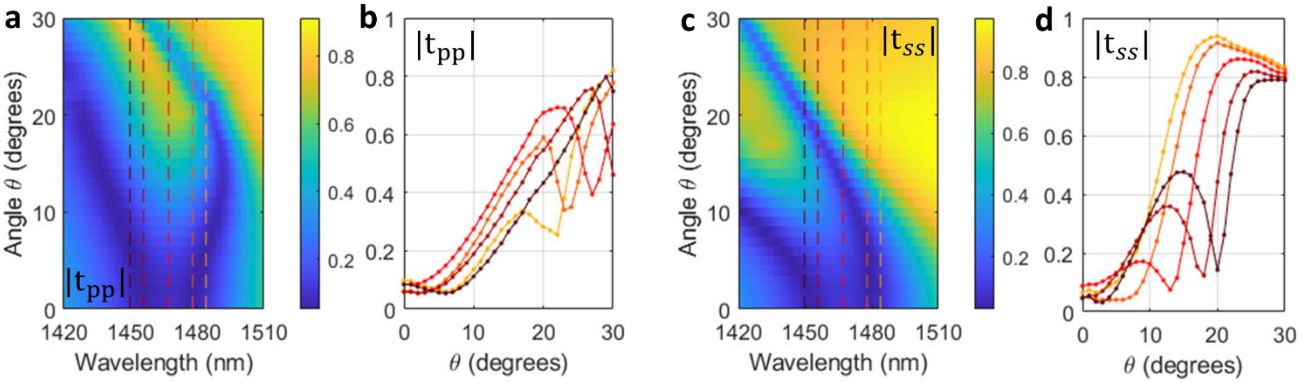

Figure S4. (a) Measured p-polarized transmission amplitude versus the polar angle θ and impinging wavelength, and for azimuthal angle $\phi = 30^o$. (b) Vertical cuts from panel a, corresponding to the color-coded dashed vertical lines. (c-d) Same as in (a-b), but for the s-polarized transmission amplitude.

S2.2 Measured transmission amplitudes for $\phi = 30^o$

Figures 2(g-j) of the main text show the measured p- and s-polarized transmission amplitudes versus wavelength and polar angle θ, for a fixed value of the azimuthal angle $\phi = 0^o$. For completeness, in Fig. S4 we show additional measured data taken at $\phi = 30^o$. These data agree very well with the corresponding simulated transfer functions (not shown here).

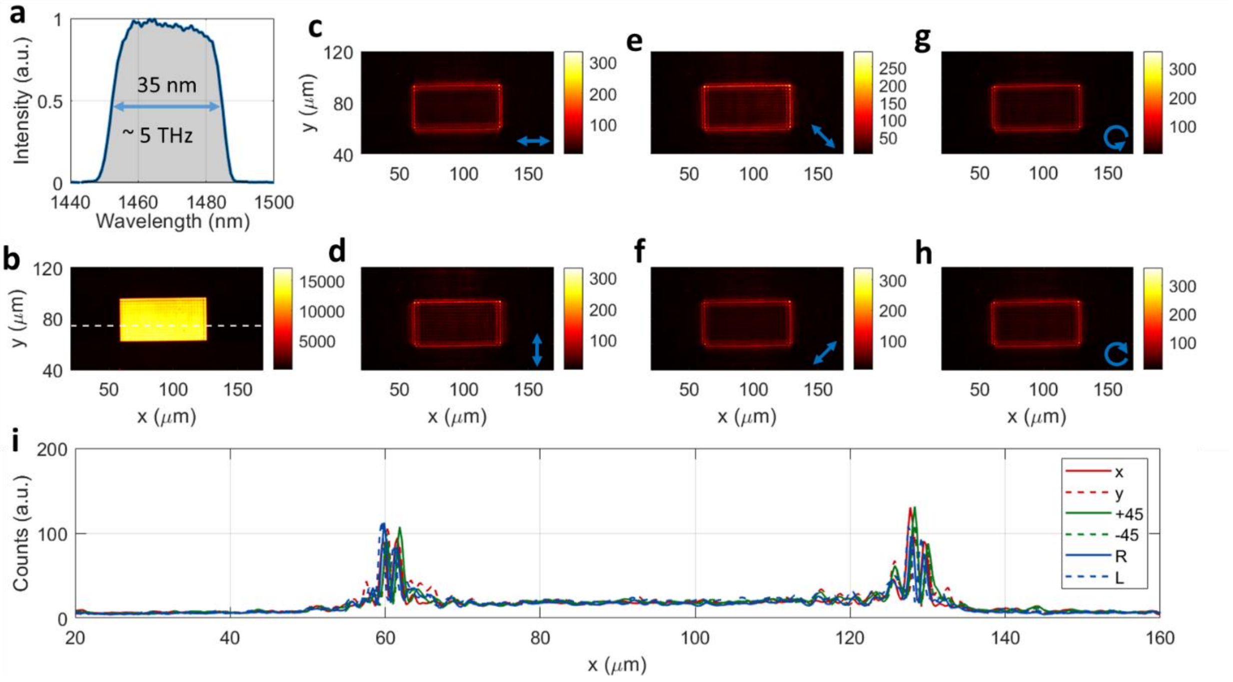

Figure S5. Edge detection with a rectangular target. (a) Spectrum of the excitation. (b) Unfiltered image. (c-h) Output images when the metasurface is placed in front of the target and for six different polarizations of the input light: linearly polarized along (c) x, (d) y, (e) the x-y diagonal, (f) the x-y anti-diagonal, or (g) right and (h) left circularly polarized. (i) Horizontal cuts of the plots in panels (c-h) (as indicated in the legend), corresponding to the vertical position denoted by the white dashed line in panel b.

S2.3 Edge detection with rectangular targets

In Fig. 5 of the main text we demonstrated broadband and polarization-independent edge detection using a CUNY logo as a test image. In Fig. S5 we show additional imaging measurements done with a rectangular shape with dimensions 70x35 µm. All experimental conditions, including the excitation spectrum, are the same as in Fig. 5 of the main text.

S3. Image processing with metasurfaces – theoretical calculations

In this paragraph we outline the mathematical steps to calculate the image processing imparted by a generic metasurface. We assume (Fig. S6) that an optical image is defined in the plane z = 0 by an intensity profile $I_{in}(x,y) = |\mathbf{E}_{in}(x,y)|^2$, where $\mathbf{E}_{in}(x,y) = E_{in}(x,y)\mathbf{e}_{in}$ is an electric field with polarization direction $\mathbf{e}_{in}$ and angular frequency $\omega = 2\pi c/\lambda = k_0 c$. For concreteness, the image can be thought as being generated by a plane wave with polarization $\mathbf{e}_{in}$ impinging on an aperture, but we notice that the calculations shown here, and the general concept of image processing, are independent of the way in which the image is created. Following standard Fourier optics [1], the image can be decomposed into a bundle of plane waves, each propagating along a direction identified by the polar and azimuthal angles θ and ϕ. In particular, assuming that the plane wave impinging on the aperture in Fig. S6 has electric field $E_0 = [E_{0,x}, E_{0,y}, 0]^T$, the field generated at a point identified by the spherical coordinates (r,θ,ϕ) is given by [2]

$$\mathbf{E}(r,\theta,\phi) = ik_0 \frac{e^{-ik_0 r}}{2\pi r} f_{in}(k_x,k_y)[\mathbf{e}_\theta(E_{0,x}\cos\phi + E_{0,y}\sin\phi) + \mathbf{e}_\phi \cos\theta\,(E_{0,y}\cos\phi - E_{0,x}\sin\phi)] \quad \text{(S1)}$$

where $f_{in}(k_x,k_y) \equiv \int dxdy e^{-i(k_x x + k_y y)} E_{in}(x,y)$ is the Fourier transform of the input image. Thus, in the far field of the image ($r \gg \lambda$), the field propagating along each direction (θ,ϕ) is given (up to an overall constant factor) by the plane wave

$$\mathbf{E}_{in}(\theta,\phi) = f_{in}(k_x,k_y)[\mathbf{e}_p E_P(\theta,\phi) + \mathbf{e}_s E_s(\theta,\phi)] \quad \text{(S2)}$$

where we identified the directional vectors of s and p polarization, $\mathbf{e}_p = \mathbf{e}_\theta$ and $\mathbf{e}_s = \mathbf{e}_\phi$, and defined $E_p(\theta,\phi) \equiv E_{0,x}\cos\phi + E_{0,y}\sin\phi$ and $E_s(\theta,\phi) \equiv \cos\theta\,(E_{0,y}\cos\phi - E_{0,x}\sin\phi)$. In Eqs. (S1)-(S2) and all equations below it is always assumed that the wave vector components $[k_x, k_y]$ depend on the angles (θ,ϕ) through the standard coordinate transfomation $[k_x, k_y] = k_0 \sin\theta[\cos\phi, \sin\phi]$. The polarization of each wave in Eq. (S2) is generally a mixture of s and p polarization, depending on the polarization of the illumination and the direction of propagation. The response of the metasurface can be

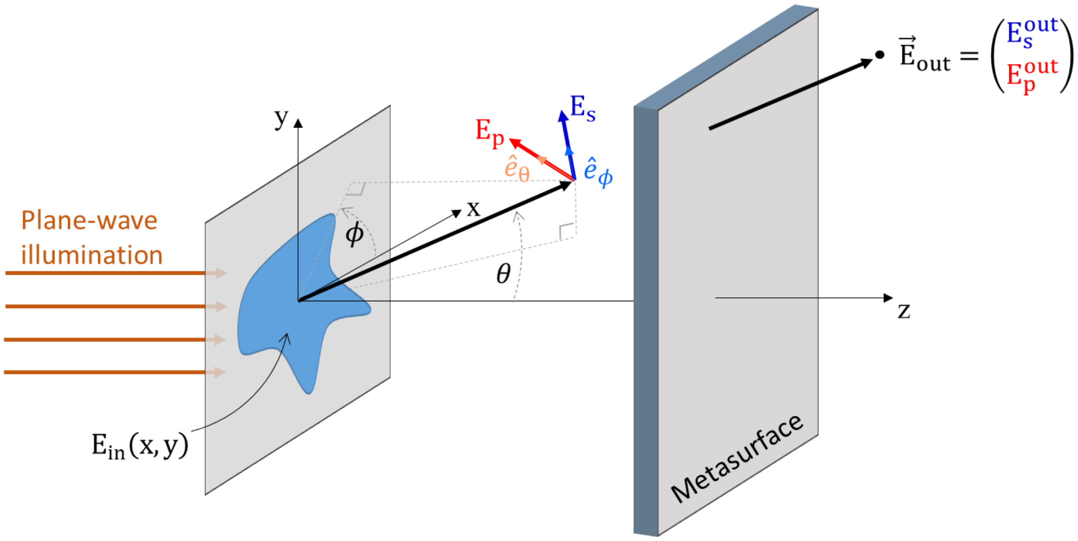

Figure S6. Schematic of the fields scattered by the image and filtered by the metasurface. See text for details.

described by a 2x2 matrix of transfer functions, describing the angle-dependent co-polarized and cross-polarized transmission coefficients

$$t(\theta,\phi) = \begin{pmatrix} t_{ss}(\theta,\phi) & t_{sp}(\theta,\phi) \\ t_{ps}(\theta,\phi) & t_{pp}(\theta,\phi) \end{pmatrix}. \quad \text{(S3)}$$

Specifically, the field transmitted through the metasurface at any given angle (θ,ϕ) is

$$\mathbf{E}_{out}(\theta,\phi) = \begin{pmatrix} E_s^{out}(\theta,\phi) \\ E_p^{out}(\theta,\phi) \end{pmatrix} = f_{in}(k_x,k_y) \begin{pmatrix} t_{ss}(\theta,\phi) & t_{sp}(\theta,\phi) \\ t_{ps}(\theta,\phi) & t_{pp}(\theta,\phi) \end{pmatrix} \begin{pmatrix} E_s(\theta,\phi) \\ E_p(\theta,\phi) \end{pmatrix} \quad \text{(S4)}$$

In order to calculate the image generated by this filtered bundle of waves, we project them back to the z = 0 plane. This is equivalent to collecting and re-focusing these waves on a plane placed at a $z = 4f$ with a pair of identical lenses with focal length f. Moreover, we transform the field into the x-y polarization basis. The overall transformation corresponds to the inverse of the plane-wave expansion in Eq. (S1). That is, apart from an overall proportionality factor,

$$\begin{pmatrix} E_x^{out}(\theta,\phi) \\ E_y^{out}(\theta,\phi) \end{pmatrix} = \overline{\overline{M}}^{-1}(\theta,\phi) \begin{pmatrix} E_s^{out}(\theta,\phi) \\ E_p^{out}(\theta,\phi) \end{pmatrix} \quad \text{(S5)}$$

where $\overline{\overline{M}}^{-1}$ is the inverse of the matrix

$$\overline{\overline{\mathrm{M}}}(\theta,\phi)=\begin{pmatrix}\cos\phi & \sin\phi\\ -\cos\theta\sin\phi & \cos\theta\cos\phi\end{pmatrix}. \tag{S6}$$

Finally, the spatially dependent output fields $E_x^{out}(x,y)$ and $E_y^{out}(x,y)$, corresponding to the electric field of the filtered image, are obtained via the inverse Fourier transform

$$E_{x/y}^{out}(x,y)=\frac{1}{2\pi}\int dxdy e^{i(k_x x+k_y y)}E_{x/y}^{out}(k_x,k_y), \tag{S7}$$

and the intensity profile is then calculated via $I(x,y)=|E_x^{out}(x,y)|^2+|E_y^{out}(x,y)|^2$. The calculations shown in Fig. 3 of the main text have been performed assuming an unpolarized excitation. To emulate the unpolarized excitation we repeated the calculations outlined above twice, assuming first an x-polarized excitation ($E_{0,x}=1, E_{0,y}=0$) and then a y-polarized excitation ($E_{0,x}=0, E_{0,y}=1$). We then averaged the filtered intensity maps $I(x,y)$ obtained in the two excitation scenarios.

Equations S4-S7 can be used to readily show that, when Eq. 1 in the main text is valid, polarization-independent second order differentiation is achieved. When Eq. 1 is valid,

$$t(\theta,\phi)=\begin{pmatrix}t_{ss}(\theta,\phi) & t_{sp}(\theta,\phi)\\ t_{ps}(\theta,\phi) & t_{pp}(\theta,\phi)\end{pmatrix}=\begin{pmatrix}C\sin^2\theta & 0\\ 0 & C\sin^2\theta\end{pmatrix}, \tag{S8}$$

the output fields in Eq. S4 become (apart from an overall constant)

$$\mathbf{E}_{out}(\theta,\phi)=\begin{pmatrix}E_s^{out}(\theta,\phi)\\ E_p^{out}(\theta,\phi)\end{pmatrix}=\sin^2\theta\cdot f_{in}(k_x,k_y)\cdot\overline{\overline{\mathrm{M}}}(\theta,\phi)\begin{pmatrix}E_{0,x}\\ E_{0,y}\end{pmatrix}. \tag{S9}$$

Thus, the filtered fields in the xy cartesian basis (Eq. S5) become

$$\begin{pmatrix}E_x^{out}(\theta,\phi)\\ E_y^{out}(\theta,\phi)\end{pmatrix}=\sin^2\theta\cdot f_{in}(k_x,k_y)\begin{pmatrix}E_{0,x}\\ E_{0,y}\end{pmatrix}. \tag{S10}$$

Finally, by applying Eq. S7 we find

$$\begin{pmatrix}E_x^{out}(x,y)\\ E_y^{out}(x,y)\end{pmatrix}\propto\nabla^2 E_{in}(x,y)\begin{pmatrix}E_{0,x}\\ E_{0,y}\end{pmatrix}. \tag{S11}$$

Equation S11 shows that any polarization of the input image (either linear, circular, elliptical or arbitrary mixture of them) will be equally processed by the metasurface, and the total spatial intensity map $I(x,y)=|E_x^{out}(x,y)|^2+|E_y^{out}(x,y)|^2$ will be independent of the input polarization.

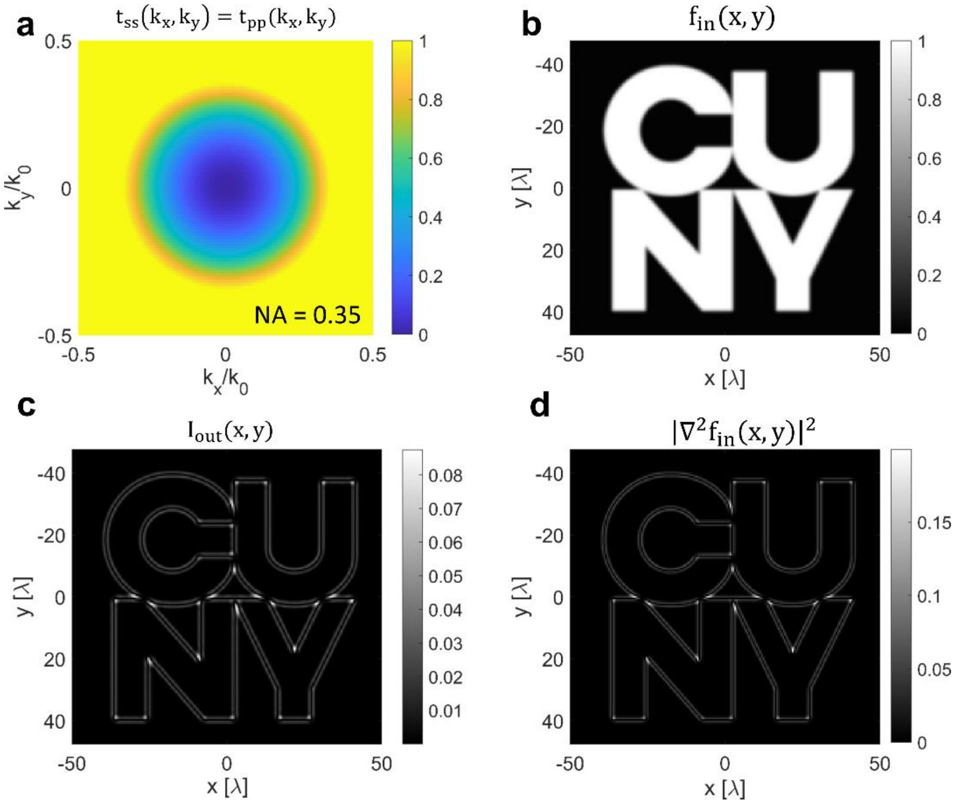

Figure S7. Calculated image processing assuming an ideal polarization-independent filter. (a) Co-polarized ideal transfer functions $t_{ss}(k_x, k_y) = t_{pp}(k_x, k_y)$ of the ideal filter (see text for details. (b) Input image. The shape and dimensions are almost identical to the target used in the experiments in Figs. 4 and 5 of the main text. (c) Output image calculated assuming the transfer function in panel a. (d) Numerically calculated Laplacian of the input image.

S4. Maximum achievable efficiency

In our experiments the peak efficiency η_{peak}, defined as the ratio between the peak intensities in the output and input images, is about 5%-10% in the monochromatic excitation case (Fig. 4 of the main text), and about 3.5% in the broadband excitation scenario (Fig. 5 of the main text). While these numbers might seem small, here we show that they are actually very close to the maximum efficiency obtainable for an ideal polarization-independent k-space filter performing edge detection at a fixed NA. To demonstrate this, we consider an ideal filter described by identical co-polarized transfer functions $t_{ss}(k_x, k_y) = t_{pp}(k_x, k_y) = t_{ideal}(k_x, k_y)$ and zero cross-polarized transfer functions, $t_{sp}(k_x, k_y) = t_{ps}(k_x, k_y) = 0$. We assume that the ideal transfer function is given by

$$t_{ideal}(k_x, k_y) = \begin{cases} \left(\frac{1}{k_0^2 NA^2}\right) \cdot \left(k_x^2 + k_y^2\right) & \text{if } k_x^2 + k_y^2 \leq k_0^2 NA^2 \\ 1 & \text{otherwise} \end{cases}.$$

This transfer function provides the required Laplacian response up to a spatial frequency corresponding to $\sqrt{k_x^2 + k_y^2} = k_0 NA$, where the transfer function reaches 1. The transmission then remains one for

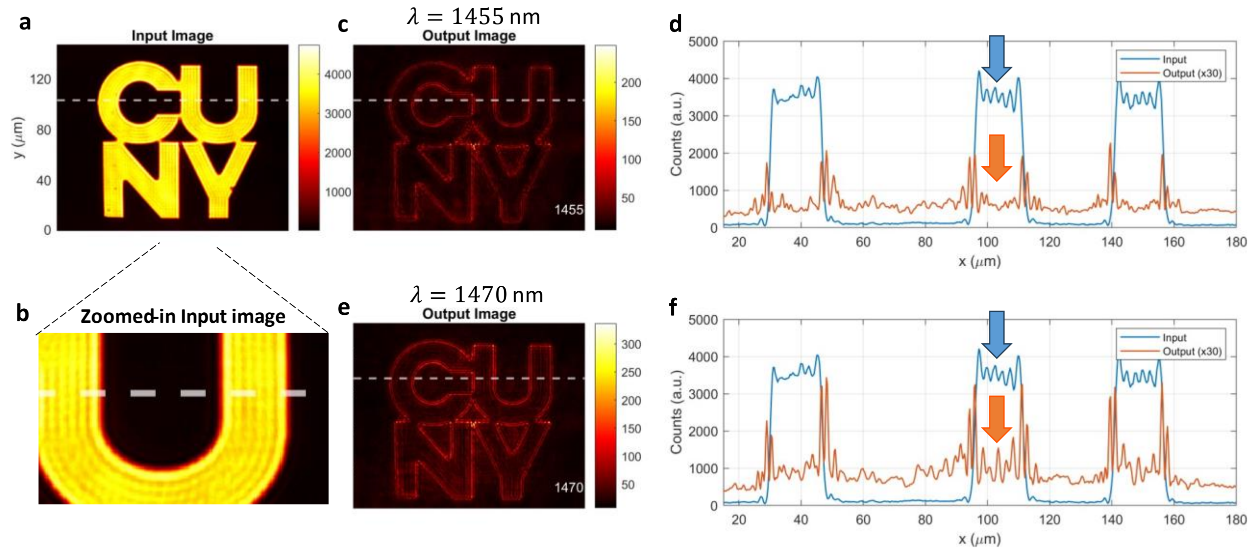

Figure S8. (a) Input image used in the experiment in Fig. 4 of the main text. (b) Zoomed-in portion of panel a, showing the weak intensity fluctuations within the bright areas. (c) Filtered image for an input wavelength of 1455 nm. (d) Horizontal cross-sectional cut of the input image (blue lines) and output image (orange lines), corresponding to the horizontal dashed lines in panels a and b. (e-f) Same as in panels (c-d) but for a wavelength of 1470 nm.

wavevectors with moduli larger than k_0NA. This device acts as an ideal edge-detectors for images whose smallest spatial features correspond to wavevectors with moduli equal to k_0NA. In Fig, S7a we consider this ideal transfer function for the case of NA = 0.35, i.e. the same NA achieved by our device experimentally. We then consider an input image (Fig. S7b) with the same shape and very similar dimensions as the one considered in the experiment, and normalized such that the maximum intensity is 1. Following the formalism described in section S4, we calculate the expected output image (Figs. S7c) assuming that the input image is processed by an ideal filter with the transfer functions shown in Fig. S7a. As expected, a clear edge enhancement occurs in the output image (Figs. S7c). The maximum intensity in the output image is about 8%-9% of the input intensity. These values matches well the typical values of 5%-10% obtained experimentally for monochromatic excitation. This shows that, for a given NA, the efficiency of our device is essentially the same as the one of an ideal edge-detector. This is also clear by comparing the output image in Fig. S7c with the image obtained by applying the exact Laplacian operator $\nabla^2 = \partial_x^2 + \partial_y^2$ to the same input image, which is shown in Fig. S7d. The peak intensity of $|\nabla^2 f_{in}(x,y)|^2$ is about 20% of the input intensity. Thus, the experimentally measured peak efficiencies $\eta_{\text{peak}} = 5\% - 10\%$ of our device are also close to the upper bound dictated by the intrinsic properties of the desired mathematical operation.

S5. Origin of additional peaks in the output images

Figure 4 and 5 of the main text show the results of the edge-detection experiments. In all experimental figures, high-intensity peaks occur at the position of the main edges of the figure, surrounded by a much weaker background. In particular, some weaker peaks are visible in the experimental plots in Fig. 4c and 5i Several effects contribute to the creation of these additional peaks and to the background, as described in this section.

While some portion of the background is due to unavoidable noise (e.g. in the camera), most of the additional peaks have actually a real physical origin, rooted in the fact that edge-detection is implemented here via the mathematical operation of Laplacian differentiation, i.e., second order derivative.

Our input images contain some "strong edges", i.e., spatial regions where the optical intensity suddenly varies from low to high values, which lead to large absolute values of the second order derivatives and thus to large-intensity peaks in the output images. In particular, each strong edge in the input image will result in two peaks in the output image, as expected from the second order differentiation of a step-like function. In a practical scenario, the presence of two peaks (instead of one) for each edge does not introduce any detrimental effect – in fact, it can be used to find the exact spatial position of each edge even more accurately.

Moreover, in our input images the intensity is not perfectly flat within the bright areas. In Fig. S8 we reproduced some of the panels of Fig. 4, together with additional cross-sectional cuts. Weak spatial fluctuations of the intensity are clearly visible both in the 2D plot in Figs. S8(a-b), and in the 1D cuts (blue lines) in Figs. S8d and S8f, denoted by the blue arrows. These spatial intensity fluctuations are due to diffraction of light at the metallic apertures that are used in our experiment to generate the input image. Since our metasurface performs a mathematical differentiation on the whole input image, these weaker spatial variations will be differentiated as well, resulting in a set of weaker peaks in the output image. Some examples of these peaks are indicated by the orange arrows in Fig. S8(d,f).

From a practical point of view, the presence of these additional weaker peaks does not limit the capability of detecting the position of the "strong edges", since the intensity of each peak in the output image is always proportional to the spatial derivative (at the same position) of the input image. Thus, the sharpest edges will always correspond to the two strongest peaks. In fact, the fact that we can experimentally detect such weak intensity fluctuations is a further demonstration of the large quality and efficiency of our metasurface.

Besides the effect discuss above, other unwanted noise and additional signals (which does not necessarily have a peak-like structure) are due to a combination of the noise in the camera used for the experiment, and of the fact that the normal-incidence transmission of the metasurface, while remaining smaller than 1%, is not exactly zero. The latter effect implies that a very small portion of the 'DC component' of the input image is transferred into the output image without any differentiation. However, as clear from the experimental data in Figs. S8(d,f) and in the main text, this effect is very small, and it does not introduce any practical issue in the edge detection.

S6. Increasing the bandwidth

In the main text we have demonstrated numerically and experimentally an edge-detecting metasurface with a bandwidth of about 5 THz around a central frequency of 198 THz. In this section, we show an example of another design, with further optimized dispersion engineering, which features a much larger bandwidth. The design has the same lattice constant as the one in the main text (a = 924 nm), while slightly different slab thickness H = 230 nm and hole radius R = 280 nm. Fig. S9a shows the normal-incidence transmission spectrum of the device (orange line), compared to the spectrum of the device considered in the main text (blue line). The two optical modes, identified by the two frequencies at which the transmission is zero, are detuned by $\Delta \approx 7.5$ THz. This, combined with increased optical linewidths, creates a range of B ≈ 10 THz where the transmission remains below 3%, ideally suited to suppress the DC component of the input image. The edge-detecting capability of this device is confirmed by the color-coded plot in Fig. S9b, which shows the p-polarized transmission versus polar angle theta at several wavelengths across the bandwidth. At all

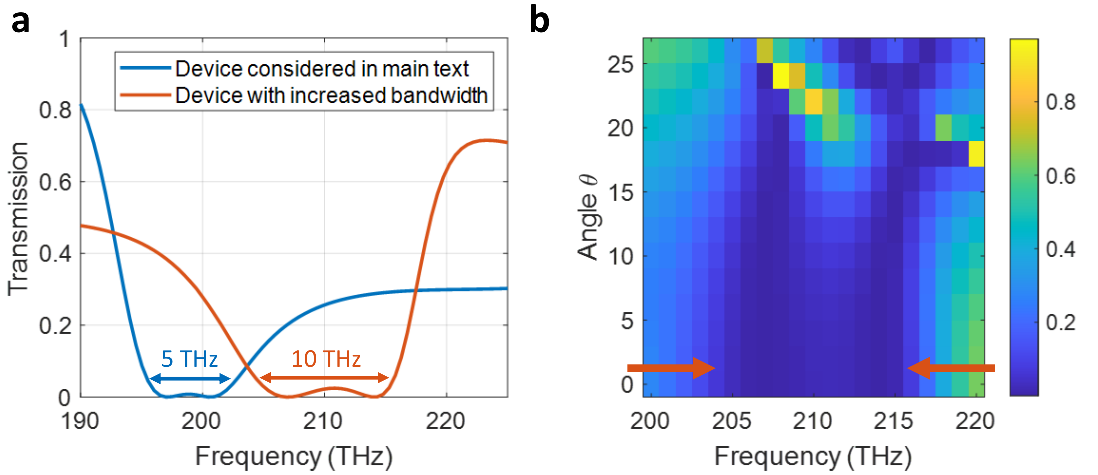

Figure S9. (a) Normal-incidence transmission spectrum of the device considered in the main text (blue line) and of a slightly different design (orange line), where the optical modes are further detuned from each other, resulting in band of about 10 THz where the transmission remains below 3%. (b) p-polarized transmission of this new design as a function of the angle θ and impinging frequency.

frequencies within a B ≈ 10 THz centered around 210 THz (68 nm at 1427 nm) the transmission is low at small angles, and it progressively increases as the angle θ increases, albeit with slightly different NAs.

Supplementary References

[1] J. W. Goodman, *Introduction to Fourier optics*, 2nd ed. New York: McGraw-Hill, 1996.
[2] R. Collin, *Antennas and Radiowave Propagation*. McGraw-Hill, 1985.
